# Difficulties in diagnosing a pediatric patient with small intestinal bacterial overgrowth

**DOI:** 10.31744/einstein_journal/2026RC2117

**Published:** 2026-02-12

**Authors:** Luanna Campos Tuñas, Ana Clara Rigueti Toma, Ricardo Katsuya Toma, Eduardo Juan Troster

**Affiliations:** 1 Hospital Israelita Albert Einstein Faculdade Israelita de Ciências da Saúde Albert Einstein São Paulo SP Brazil Faculdade Israelita de Ciências da Saúde Albert Einstein, Hospital Israelita Albert Einstein, São Paulo, SP, Brazil.; 2 Universidade de São Paulo Faculdade de Medicina São Paulo SP Brazil Faculdade de Medicina, Universidade de São Paulo, São Paulo, SP, Brazil.

**Keywords:** Gastrointestinal microbiome, Bacteria, Dysbiosis, Intestine, small, Child, preschool

## Abstract

A 3.5-year-old female presented with a 10-month history of abdominal pain and distension, accompanied by frequent belching and flatulence. No nausea, vomiting, or nighttime awakening due to pain was reported, and the patient's appetite remained intact. Growth and development were age-appropriate (weight: 16.2kg, height: 106cm). She was born at term without complications and had a normal neonatal screening. No comorbidities typically associated with small intestinal bacterial overgrowth (SIBO)—such as prior intensive care unit admission, immunodeficiency, food intolerance, *Helicobacter pylori* infection, anatomical abnormalities, prior abdominal surgeries, motility disorders, autoimmune conditions, or hepatic condition—were identified. Initial diagnostic hypotheses included more prevalent gastrointestinal disorders such as lactose intolerance, celiac disease, intestinal parasitosis, chronic constipation, and intestinal malformations (*e.g*., intestinal malrotation). SIBO was suspected following a thorough reassessment of the clinical history. The diagnosis was confirmed via a hydrogen breath test with lactulose, supported by radiological findings. Following treatment with metronidazole (30 mg/kg/day for 10 days), the patient demonstrated significant clinical resolution of the previously reported symptoms. A follow-up hydrogen breath test revealed no further evidence of bacterial overgrowth in the small intestine.

## INTRODUCTION

The small intestine (SI) is inhabited by bacteria that form part of the human microbiota; however, compared with the colon, the quantity and function of the bacterial population in the SI remain poorly understood.^(1)^ Small Intestinal Bacterial Overgrowth Syndrome (SIBO) is a gastrointestinal condition characterized by an imbalance of the intestinal microbiota resulting from increased bacterial counts in the upper gastrointestinal (GI) tract.^(2)^ This dysbiosis, which is scarcely described in the literature, may cause gastrointestinal symptoms such as bloating, early satiety, abdominal pain, nausea, flatulence, loss of appetite, diarrhea, constipation, and malnutrition,^(3)^ although it may also be asymptomatic. Patients with SIBO typically exhibit increased levels of *Klebsiella, Escherichia/Shigella,* and *Acinetobacter* species.^(2)^

The composition of the intestinal microbiota is influenced by factors such as age, sex, diet, nutritional habits, and immune status.^(4)^ The stomach's acidic pH protects against bacterial overgrowth in the upper GI tract.^(5)^ Epidemiologically, SIBO is associated with patients in intensive care units,^(4)^ chronic proton pump inhibitor (PPI) use,^(6)^ and *Helicobacter pylori* infection,^(5)^ but is also prevalent—though underdiagnosed—in nonsurgical patients with intestinal dysmotility and impaired protective mechanisms that regulate bacterial proliferation.^(2)^ Major risk factors include GI anatomical changes (most significant), prior abdominal surgeries, postsurgical adhesions, ileocecal resections, constipation from various causes, liver disease, autoimmune gastritis, Parkinson's disease, and chronic pancreatitis.^(2)^

Early diagnosis of SIBO is hindered by the wide range of nonspecific symptoms and the limited accuracy of currently available diagnostic techniques. The diagnostic gold standard involves duodenal or jejunal aspirate cultures obtained via endoscopy, a procedure that is invasive and time-consuming. Breath tests based on hydrogen and methane measurements in exhaled air following carbohydrate ingestion are more practical and are recommended as the initial diagnostic screening tests for SIBO.^(7)^

There is no established gold-standard treatment for SIBO; antibiotics are commonly used for symptom relief,^(8)^ often in combination with dietary modifications.^(2)^ When SIBO is secondary to an underlying condition, management should focus on addressing the primary cause.

In children, SIBO remains underdiagnosed, and a high index of clinical suspicion—based on careful evaluation and detailed history-taking—is essential for diagnosis. Given the scarcity of studies on this topic, this report presents a clinical case of SIBO in a child and discusses its diagnosis, clinical management, and follow-up.

## CASE REPORT

A 3.5-year-old girl presented with abdominal pain and distension lasting 10 months, accompanied by frequent belching and flatulence. The symptoms began insidiously and gradually progressed. Seven months prior to presentation, the patient experienced two episodes of acute otitis media that were treated with amoxicillin-clavulanic acid. Following antibiotic administration, progressive worsening of abdominal distension, flatulence, and belching was observed, accompanied by episodes of periumbilical abdominal pain, particularly after meals. The mother reported worsening of symptoms with cow's milk products, particularly those high in lactose, as well as sweet foods. Symptomatic improvement was noted following passage of flatus and the use of simethicone and common analgesics. No nausea, vomiting, or nighttime awakening due to pain was reported, and the patient's appetite remained good. However, due to pain, she was frequently picked up from school and avoided extracurricular activities. The mother also reported hard stools (Bristol stool types 1 and 2), occurring daily or every other day, beginning after toilet training at age 2. The caregiver observed frequent episodes of fecal retention. The patient's diet was imbalanced, characterized by excessive consumption of sweet and confectionery products. The child was up to date on vaccinations and had no relevant family history.

Growth and development were appropriate for age (weight, 16.2kg; height, 106cm). The patient was born at term via caesarean section, with no complications, and had normal neonatal screening results. On examination, the patient was in good general condition, with no peripheral edema, good perfusion, and symmetric peripheral pulses. No cardiovascular, genitourinary, dermatologic, or neurologic abnormalities were observed. No otorhinolaryngologic abnormalities were observed. The abdomen was globose, tense, distended, and nontender on both superficial and deep palpation, with a nonpainful rebound, tympanic percussion in all quadrants, and audible bowel sounds with borborygmi.

**Figure 1:**
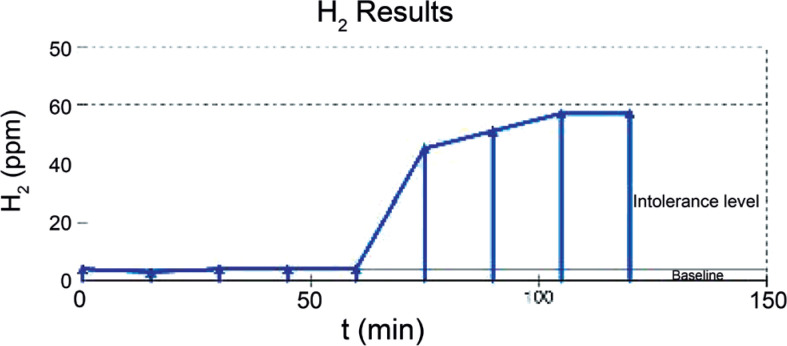
Expired hydrogen breath test results, in which analysis of the H_2_ curve demonstrated early bacterial metabolism of lactulose (bacterial overgrowth) beginning 60 minutes after substrate ingestion, establishing the diagnosis of SIBO

**Figure 2:**
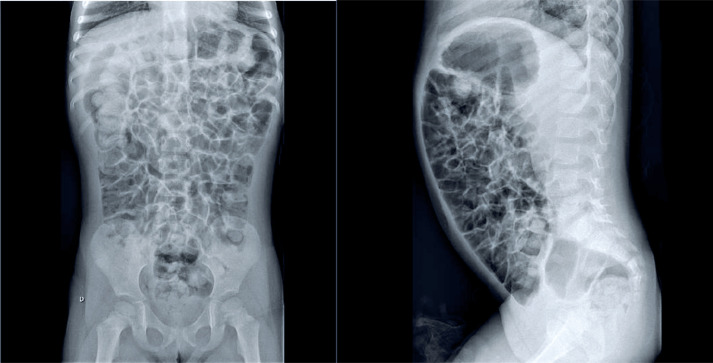
Abdominal X-rays showing mild diffuse gaseous distension of colonic and small bowel loops without pathological calcifications

The differential diagnoses included lactose intolerance, celiac disease, intestinal parasitosis, chronic constipation, and intestinal malrotation. Intestinal transit radiography ruled out structural abnormalities or malformations. Lactose intolerance was excluded due to lack of symptom resolution following dietary elimination. Parasitological examination results were negative. Serologic testing for anti-tissue transglutaminase antibodies and total IgA levels ruled out celiac disease. Constipation was treated with polyethylene glycol, without clinical improvement. The immune function was evaluated and found to be normal. Therefore, after reassessing the clinical history of flatulence and physical examination findings revealing abdominal distension and pronounced hydro-aerial sounds, SIBO was considered a possible cause of the abdominal distension. A hydrogen breath test with lactulose (Figure 1) was performed following oral administration of lactulose at a dose of 0.5g/kg body weight, diluted in 100mL of water. Expired hydrogen (H_2_) levels were measured every 15 minutes using a Hydra MicroH_2_ Breath Analyzer (Micro Medical Limited). The results were expressed in parts per million (ppm). Analysis of the H_2_ curve demonstrated early bacterial metabolism of lactulose, consistent with bacterial overgrowth, beginning 60 minutes after substrate ingestion. This finding confirmed bacterial colonization of the small intestine, thereby establishing the diagnosis of SIBO. Abdominal radiography (Figure 2) revealed mild, diffuse gaseous distension of the colonic and small bowel loops, with no pathological calcifications.

Initial management included dietary modifications and treatment of constipation with polyethylene glycol. Despite correction of these factors, gastrointestinal symptoms persisted. Pharmacological treatment was initiated with metronidazole at a dose of 30mg/kg/day for 10 days. Following the course of metronidazole treatment, the patient demonstrated significant clinical improvement in previously reported symptoms, with the exception of intermittent abdominal discomfort. Follow-up hydrogen breath testing revealed no evidence of SIBO.

## DISCUSSION

Small intestinal bacterial overgrowth typically presents with non-specific signs and symptoms, complicating the diagnostic process due to its clinical overlap with more prevalent conditions. Consequently, the diagnosis of SIBO is often delayed, as observed in the current case.

The patient presented with frequent belching, flatulence, postprandial abdominal pain, and abdominal distension; however, she did not experience nausea or diarrhea, symptoms characterized by Stachowska et al. as typical of SIBO.^(3)^ She also presented with constipation and abdominal bloating, though her appetite remained intact.^(3)^ These symptoms overlap significantly with common gastrointestinal disorders such as lactose intolerance, celiac disease, intestinal parasitosis, chronic constipation, and intestinal malformations, all of which were initially considered in the differential diagnosis.

However, unlike typical clinical presentations, this patient lacked most of the recognized risk factors that prompt a suspicion of SIBO; she had no history of Intensive Care Unit admission, immunodeficiency, or food intolerance. Furthermore, she presented no anatomical abnormalities, prior abdominal surgeries, autoimmune gastritis, intestinal dysmotility, chronic pancreatitis, Parkinson's disease, or hepatic conditions, such as steatosis. In summary, the patient had no prior comorbidities, supporting the diagnosis of SIBO.

The patient had no history of using proton pump inhibitors (PPIs) for abdominal pain management—a therapy identified by Sharabi et al. as a potential, albeit controversial, risk factor.^(2)^ PPI use can reduce gastric acidity and alter intestinal motility, potentially facilitating bacterial overgrowth, as demonstrated by Durán-Rosas et al.^(6)^

While there is no direct evidence linking antibiotics such as amoxicillin-clavulanic acid to SIBO, these agents can alter gut microbiota composition and may compromise protective mechanisms that regulate bacterial proliferation, as mentioned by Sharabi et al.,^(2)^ together with bowel dysmotility.

Small intestinal bacterial overgrowth was diagnosed using the hydrogen breath test with lactulose, the most commonly used noninvasive diagnostic modality in pediatrics, offering 60-70% sensitivity and 40-80% specificity.^(9)^ The gold standard remains duodenal aspirate culture on MacConkey agar (threshold >10³CFU/mL); however, this method is invasive, costly, and carries anesthesia-related risks. Additionally, aspirate cultures are susceptible to false negatives in cases involving the distal small bowel.^(2)^ Thus, the hydrogen breath test remains a more practical and widely used alternative, particularly in pediatric populations. The hydrogen breath test has significant limitations in diagnosing SIBO in children. These include low sensitivity and specificity, a lack of universal standardization of protocols, and challenges in interpretation owing to variable substrate absorption and intestinal transit times. The American College of Gastroenterology notes that glucose breath tests may miss distal SIBO because glucose is absorbed proximally; conversely, lactulose breath tests are prone to false positives due to rapid transit and colonic fermentation, especially in children with motility disorders or altered anatomy.^(10,11)^ There are also inconsistencies in diagnostic thresholds and timing, and the presence of methanogens can suppress hydrogen production, further complicating interpretation.^(10,12–13)^ Variability in preparation and performance, as well as the influence of recent antibiotics, diet, and medications, can affect the results.^(10,12–14)^ Novel capsule-based diagnostic approaches show promise for future noninvasive, accurate assessment.^(10,15)^

Small intestinal bacterial overgrowth treatment focuses, where applicable, on resolving predisposing causes. In this case, since comorbidities were absent, treatment was administered with metronidazole, a broad-spectrum antibiotic effective against anaerobes. Medical literature highlights that, while antibiotics are the cornerstone of therapy, there is no standardized regimen, dosing, or duration for pediatric SIBO; most recommendations are extrapolated from adult studies or based on expert opinions and small case series.^(10,15–20)^ Oral metronidazole was chosen for this case because it is a commonly used, effective, and accessible empiric agent, especially in pediatric and intestinal rehabilitation settings. Rifaximin, per Sharabi et al., eradicated symptoms in 50% of patients after 14 days;^(2)^ however, pediatric data remain limited, and no single antibiotic has been proven superior in children.^(8)^ Both metronidazole and rifaximin are frequently used; however, international survey data indicate that metronidazole is the most common first-line agent among pediatric intestinal rehabilitation and nutrition support providers, with rifaximin being the second most common choice.^(16)^ This practice pattern reflects providers’ familiarity, accessibility, and cost considerations. In the Brazilian context, rifaximin is a high-cost medication that may be prohibitively expensive and less readily available; consequently, metronidazole was selected as the initial treatment.^(12, 21)^ A retrospective cohort study by Peinado Fabregat et al. found no difference in efficacy between metronidazole and rifaximin, though the study had limitations, including a small sample size.^(9)^ The article by Silva refers to the use of antibiotics as the primary component of treatment, along with the adoption of a low-FODMAP (Fermentable Oligosaccharides, Disaccharides, Monosaccharides, and Polyols) diet for six weeks to reduce symptoms.^(22)^ Important gaps in evidence remain, with a clear need for robust pediatric trials and consensus guidelines to inform optimal antibiotic selection, dosing, and duration for SIBO in children.^(15,16,18)^ Until such data are available, the choice of metronidazole as first-line therapy in pediatric SIBO is supported by prevailing clinical practice, published efficacy data, and practical considerations of cost and accessibility. However, consistent studies on the use of probiotics are still lacking**.**

## CONCLUSION

This case report emphasizes the need for greater awareness of Small Intestinal Bacterial Overgrowth Syndrome in clinical practice, especially among pediatricians, to prevent diagnostic delays and improve patient quality of life. Small intestinal bacterial overgrowth syndrome presents with diverse and nonspecific symptoms that lead to frequent underdiagnosis, largely due to its clinical overlap with more prevalent gastrointestinal diseases. To reinforce the clinical takeaway from this case, small intestinal bacterial overgrowth syndrome should be considered in pediatric patients presented with persistent, nonspecific gastrointestinal symptoms, even in the absence of traditional risk factors or typical comorbidities.

## Data Availability

The underlying content is contained within the manuscript.
